# Body Positivity and Self-Compassion on a Publicly Available Behavior Change Weight Management Program

**DOI:** 10.3390/ijerph182413358

**Published:** 2021-12-18

**Authors:** Meaghan McCallum, Annabell Suh Ho, Christine N. May, Heather Behr, Ellen Siobhan Mitchell, Andreas Michealides

**Affiliations:** 1 Academic Research, Noom, 229 W 28th St., New York, NY 10001, USA; meaghanm@noom.com (M.M.); annabell@noom.com (A.S.H.); christinem@noom.com (C.N.M.); hbehr@saybrook.edu (H.B.); andreas@noom.com (A.M.); 2 Department of Integrative Health, Saybrook University, 55 W Eureka St., Pasadena, CA 91103, USA

**Keywords:** obesity, weight loss, weight management, mHealth, digital health, body positivity, self-compassion, CBT, ACT, body image, body dissatisfaction

## Abstract

According to recent research, body positivity and self-compassion are key outcomes that are tied to better psychological and physical health. To date, it is unclear whether body positivity and self-compassion improve, stay constant, or deteriorate over the course of a weight management program, particularly one that addresses the psychological roots of behavior change. Additionally, beyond controlled settings, there are no studies on body positivity and self-compassion in individuals who choose to join a commercial weight management program. Therefore, this single-arm prospective study examined changes in body positivity and self-compassion from baseline to the 16 week milestone of Noom Weight, a commercial behavior change weight management program informed by acceptance and commitment therapy (ACT), dialectical behavior therapy (DBT), and cognitive behavioral therapy (CBT). We also examined how baseline and over-time changes in body positivity and self-compassion predicted engagement in program-measured relevant behaviors (e.g., exercises logged). Participants were a random subset of individuals who had recently self-enrolled in the program (*n* = 133). Body positivity and self-compassion were measured via survey at baseline and end of the core program (16 weeks). Self-reported weight and program-recorded engagement were extracted from the program database. Compared to baseline, body appreciation, body image flexibility, self-compassion, and body-focused rumination significantly improved at 16 weeks (all *p*s < 0.007). Participants lost a statistically significant amount of weight (3.9 kg; t(128)) = 10.64, *p* < 0.001) by 16 weeks, which was 4.4% body weight. Greater engagement, especially messaging a coach, reading articles, and logging meals, was associated with improvements over time in body appreciation (r = 0.17, *p* = 0.04), body image flexibility (r = −0.23, *p* = 0.007), and the brooding component of rumination (r = −0.23, *p* = 0.007). Greater engagement was also associated with baseline total self-compassion (r = 0.19, *p* = 0.03) and self-judgment (r = 0.24, *p* = 0.006). The results suggest that individuals experience improvements in body positivity and self-compassion while learning about ACT, DBT, and CBT through curriculum and coaching in this setting. The results also have important clinical implications, such as the possibility that psychologically-oriented (i.e., ACT, DBT, and CBT-based) weight management could be important to improve body positivity or that baseline self-compassion could be used to target individuals at risk for lower engagement. Future work should investigate these possibilities as well as delineate the causal relationships between body positivity, self-compassion, engagement, and weight loss.

## 1. Introduction

A positive body image and self-compassion are associated with greater psychological well-being and engagement in healthy behaviors [1,2,3,4,5]. Recent scholarship has conceptualized a positive body image, or body positivity, as a positive orientation towards one’s body, distinct from low body dissatisfaction or negative body image. A positive body image includes body acceptance, or accepting one’s body as it is, and body image flexibility, or non-judgmentally accepting negative body-related emotions or experiences and then engaging in meaningful behaviors [3,6]. Self-compassion is extending kindness and understanding to oneself or one’s distressing experiences, thoughts, or feelings. Research has increasingly found that self-compassion is strongly linked to body positivity [7,8]. For example, individuals report better body positivity on days they show themselves more self-compassion [9].

An important question relates to individuals’ body positivity and self-compassion on weight management programs. Most studies have examined standard behavioral weight (SBW) programs, which provide practical support and tools aimed to help individuals achieve goals of moderate weight loss. These studies have primarily examined negative body image rather than body positivity, and findings are mixed. According to recent systematic reviews of mostly randomized controlled trials, some studies have shown no change in body image or body dissatisfaction, other studies have shown improvements, and still other researchers have expressed concern about potential negative effects from monitoring one’s weight [10,11,12]. Additionally, researchers have noted that SBW programs insufficiently address underlying psychological factors, such as relationships towards food or negative emotions and thoughts towards one’s body or self [13,14,15].

There is reason to believe that programs informed by acceptance and commitment therapy (ACT), dialectical behavior therapy (DBT), or cognitive behavioral therapy (CBT), could lead to more consistent improvements in body positivity and self-compassion than those demonstrated in SBW programs so far. ACT and DBT aim to help individuals accept and integrate distressing emotions, thoughts, and experiences, and to take meaningful action [16,17,18]. For example, ACT could help an individual to become non-judgmentally aware of aversive negative thoughts and fears surrounding their body and how it may be perceived, identify value-laden motivations for healthy behaviors (e.g., running with my grandchildren vs. looking good), and to process negative thoughts in ways that may encourage meaningful action (e.g., going to the gym) [18]. In addition to helping with body positivity, these principles could help individuals to extend themselves self-compassion. They could help individuals to give themselves kindness and compassion in the midst of distressing thoughts or emotions, learn that they do not need to blame themselves for not being perfect, and become non-judgmentally aware of emotions and thoughts just as they are [19]. Further, CBT is designed to help individuals modify negative thought patterns, such as ruminative negative thoughts [15,20]. ACT-, DBT-, or CBT-informed programs that primarily target body image, eating disorder prevention, or self-compassion have been found to improve body dissatisfaction or self-compassion [19,21,22,23,24,25]. Studies of generalized programs (i.e., not specifically targeted for body image or self-compassion, but with some psychological component) have shown improvements in psychological well-being as well as body image [26,27]. However, the question still remains whether improvements in body positivity and self-compassion occur on generalized weight management programs informed by ACT, DBT, or CBT.

Therefore, this study aims to contribute to the literature in four major ways. First, almost all previous studies were in controlled settings (e.g., controlled trials) with individuals who were recruited to take part in this study. However, millions of individuals with obesity voluntarily join digital commercial programs outside the context of a controlled trial and self-manage their own participation. There is almost no relevant work on this type of program, though it is a different setting (i.e., self-managed vs. researcher-managed) with a potentially different population [28]. For example, it is possible that individuals who choose to join a publicly available program show different changes over time in body positivity or self-compassion than individuals recruited for controlled trials. Therefore, it is important to contribute to existing knowledge with data from this type of program. Another gap in the literature is that there is little knowledge about weight management programs and body positivity, rather than body image or body dissatisfaction. As explained previously, some studies have shown that body image and body dissatisfaction can improve on weight management programs over time [10]. However, it is conceivable that weight management programs could improve body dissatisfaction, but not necessarily improve *positivity* and acceptance of one’s own body, making it important to explore body positivity as an outcome. There is also little current knowledge of how generalized weight management programs improve self-compassion. Additionally, while improvements have been reported in psychologically-oriented (e.g., CBT-based) programs *focused* on body image or dissatisfaction, it is unclear whether CBT-based *weight management* programs can improve body positivity. This study adds to current knowledge by providing data on changes in body positivity and self-compassion on a CBT-based weight management program. Finally, there is little, if any, work on how baseline body positivity and self-compassion, or changes in those factors over time, predict real-world engagement in relevant behaviors (e.g., food or exercise logged).

Therefore, in this single-arm prospective study, participants who had self-enrolled in a digital commercial behavior change weight loss program informed by ACT, DBT, and CBT were followed from baseline to end of the program with data extracted from the program database and self-reported by survey. This provided the unique opportunity to examine changes in body positivity and self-compassion in a setting that reflects real-world use of the program as much as possible (i.e., with individuals who self-voluntarily joined the program and minimal participation requirements). For this study’s primary aim, based on past work on ACT, DBT, and CBT [19,21,22,23,24,25], we hypothesized that body acceptance, body image flexibility, and self-compassion would significantly increase from baseline to end of the program and body-focused rumination (i.e., continuous negative thought patterns) would significantly decrease. We also hypothesized that these improvements would be independent of weight lost, with no significant associations between weight loss and body appreciation, body image flexibility, self-compassion, and body-focused rumination. The secondary aim was to explore associations between engagement and baseline as well as over-time changes in body acceptance, body image flexibility, self-compassion, and body-focused rumination.

## 2. Materials and Methods

This study was a single-arm prospective cohort design. A prospective design helps to minimize bias when observing changes and ensure that outcomes occurred *over time* and that baseline measures could be used to predict future changes. We chose a single-arm design because the motivation for this study was to understand the outcomes that occurred *on* this type of program, and among a population motivated to self-enroll in a real-world program.

### 2.1. Participants

**Figure 1:**
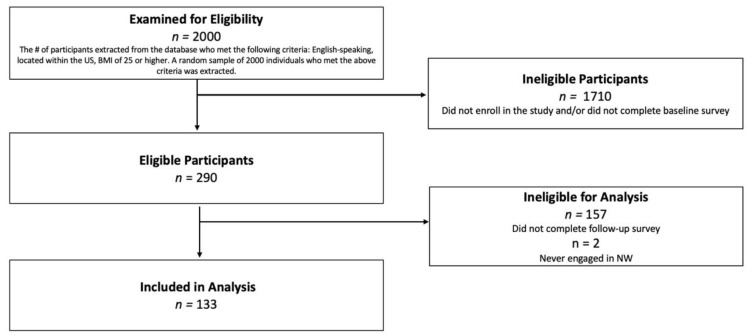
Diagram of study inclusion.

Participants included adults who voluntarily signed up for Noom Weight (NW) during a one-week period in March 2021. A random subset of 2000 users who elected to subscribe to NW following a two-week trial period were invited to participate in this prospective study. Inclusion criteria assessed at signup were: located within the US, BMI of 25 or higher, and English speaking. A total of 290 NW users enrolled in the current study and completed a baseline survey. Of these participants, 135 also completed a follow-up survey at week 16. Two participants who never engaged in NW were excluded from analysis, resulting in a final sample of 133 participants who completed both surveys (see Figure 1). The 133 participants who completed both baseline surveys did not significantly differ in any demographic variable or baseline outcome from the individuals who completed only the baseline survey.

### 2.2. Power and Sample Size Calculation

An a priori sample size calculation was performed with G*Power for our primary aim. Regarding our hypothesized difference in body positivity from baseline to follow-up, power was calculated based on the within subjects effect of a short-term, CBT-based weight loss intervention on body satisfaction reported by Rapoport and Wardle [29] (d = 0.55; 2000). Using an alpha error probability of 0.05, the total sample size needed for a paired samples t test to achieve a corresponding power of 0.95 was estimated to be 45 participants. Thus, the final sample size of 133 was considered to be adequately powered.

### 2.3. Procedure

This study was approved by the Advarra Institutional Review Board. A random subset of NW subscribers who signed up for the program between 24 February and 20 March 2021 were invited to participate in the current study. As part of the approved consent process, all potential participants were given the option to opt in to prospective research studies or the option to opt out. In opting in, participants agreed to the use of their de-identified program data for research purposes, and were told they could opt out at any time. Participants were invited to complete the baseline questionnaire following the 2 week trial period. Those who completed the baseline questionnaire were invited to participate in the follow-up survey after a cumulative 16 weeks using NW. A duration of 16 weeks was chosen because that is the length of the core curriculum before individuals are introduced to maintenance material. Engagement with in-app actions encouraged by the program (e.g., reading articles) was directly recorded within the program. Study completers were compensated for their participation. In order to measure real-world engagement, participants were not given specific minimum engagement requirements to stay in this study.

### 2.4. Program

Noom Weight is a mobile health behavior change program that has demonstrated effects on clinically significant weight loss [30,31,32,33]. NW leverages evidence-based techniques in behavior change and weight control, drawn from ACT, DBT, and CBT. For example, the curriculum introduces the framework (e.g., what is DBT) and describes its principles (e.g., components of DBT such as mindfulness, emotional regulation, and distress tolerance), explains how these principles can help, and provides practical tips on how to incorporate these principles or understanding into users’ daily lives (e.g., “Close your eyes and visualize your breath traveling in and out as you breathe”). The curriculum covers primary components of ACT, DBT, and CBT, such as accepting negative emotions, feelings, and thoughts; mindfulness; acceptance-based integration and movement towards value-laden meaningful behaviors; and recognizing, unpacking, and replacing thought distortions or ruminative thinking [34,35,36]. Articles also include principles of self-compassion, and explain the importance of showing oneself kindness and understanding even with limitations or failures and refraining from self-judgment. In terms of body positivity, articles explain possible sources of body image, help users to reflect on what their body image truly is and where it came from, and encourage users to reflect on thoughts that appreciate their body or restructure thoughts towards self-acceptance of one’s body and away from self-value based on appearance. The curriculum also includes aspects of psychological well-being such as stress management, coping skills, goal-setting, and cognitive flexibility. Mobile features enable hands-on activities and reflection; for example, in CBT-informed articles on thought distortions and ruminations, users are guided through writing down their thoughts and emotions, providing supporting and contrary evidence, and describing the objective facts of the situation to identify distortions, with their previous responses displayed at key moments of reflection.

Users self-enroll in the mobile program by downloading it on their smartphone from the app store or via the Noom website. Within the first few days of the program, users are guided through an exercise in which they set an overall goal for their participation in the program. Users are guided away from weight loss goals (e.g., lose 20 pounds) and towards value-laden goals (e.g., have energy for my grandchildren) with sequential “why?” questions. Users are then reminded of these value-laden goals periodically throughout the program. On the mobile program, users are provided access to food, exercise, and weight logging features; individual and group coaching; and an interactive curriculum, all of which all have been shown to be effective components of weight management interventions [37,38]. In addition to the psychologically-informed content described above, the curriculum involves daily articles on empirically-supported psychological principles, nutrition, physical activity, and behavior change. Coaches, trained to use techniques from CBT and motivational interviewing, interact with users via in-app text messaging to help emphasize psychological principles, to help users to set and meet goals, and to help users reflect on their progress [39]. Logging features allow users to input the foods they have eaten for a given meal, their weight, or the type of exercise they did each day, in order to self-monitor their patterns and progress. Users are encouraged but not required to log their meals and weight daily, and logging fear and avoidance are progressively addressed throughout the curriculum. When logging foods, users can choose from a food database containing thousands of foods or users can input their own custom entry. Users have access to an analysis showing them the percentage of high-calorie-dense, medium calorie-dense, and low calorie-dense foods they have eaten so far each day. The program recommends a ratio of 25%, 35%, and 45%, respectively. Adhering to the recommended ratios is associated with greater weight loss [40]. In the latter half of the program, users are put into online groups with other users. Users can add posts or react to others’ posts with comments or reactions (i.e., hearts).

### 2.5. Measures

Identical surveys were completed at baseline and the 16 week follow-up periods, with the addition of demographic information assessed at baseline. For each self-report, change scores were computed by subtracting baseline scores from week 16 scores. For all of these survey measures, there were no missing data.

Body Appreciation Scale-2 (BAS). The BAS [41] assessed the degree to which participants held an accepting and positive view of their body’s features, regardless as to if they also held a certain degree of body dissatisfaction. This 10-item measure yielded an overall average body appreciation score. Internal reliability (α = 0.93 and 0.94 for baseline and follow-up, respectively) and the stability coefficient (r = 0.78) reflected good reliability.

The Body Image-Acceptance and Action Questionnaire (BI-AAQ). The BI-AAQ [42] assessed the degree to which participants endorsed body image flexibility: the capacity to experience one’s perceptions, thoughts, and feelings associated with one’s body fully, even when difficult, while simultaneously and intentionally engaging in behavior that is consistent with one’s chosen values. Body image flexibility is not characterized by the specific content of thoughts (e.g., “What I look like is an important part of who I am”), but on how body image is impacting life (e.g., “When I start thinking about the size and shape of my body, it’s hard to do anything else”). This 12-item measure yielded a total body image flexibility score. Internal reliability (α = 0.90 for both baseline and follow-up) and the stability coefficient (r = 0.73) reflected good reliability.

Self-Compassion Scale, Short Form (SCS-SF). The SCS-SF, a well-validated measure of self-compassion [43], assessed participants’ ability to hold one’s feelings of suffering with a sense of warmth, connection and concern. This 12-item measure yielded a total self-compassion score in addition to six subscale scores: (1) self-kindness and (2) self-judgment: the ability to treat oneself with care rather than harsh self-judgment; (3) common humanity and (4) isolation: the ability to recognize that imperfection is a shared aspect of the human experience rather than feeling isolated; and (5) mindfulness and (6) over-identification: the ability to hold one’s experience in balanced perspective rather than exaggerating the storyline of suffering or overidentifying with emotions. Internal reliabilities ranged from α = 0.57 to 0.89 and stability coefficients ranging from r = 0.56 to 0.78, indicating adequate reliability.

Ruminative Response Scale for Eating Disorders (RRS-ED). The RRS-ED [44] assessed participants’ mental ruminations concerning food, figure and weight. This scale was originally validated on healthy (i.e., without eating disorder) individuals to understand body-focused rumination [44]. This 9-item measure yielded a total rumination score in addition to two subscale scores: (1) brooding: the tendency to compare one’s recent eating-related behavior, figure, or weight with some ideal and (2) reflection: the tendency to deliberately think about one’s eating, weight, and/or shape. Internal reliabilities ranged from α = 0.60 to 0.89 for baseline and follow-up subscale and overall rumination scores. Stability coefficients ranging from r = 0.55 to 0.73, indicating adequate reliability.

Noom Engagement. On Noom, individuals are encouraged, but not required, to read articles, log their food, weight, and exercise; message their coach; and post on online groups. Engagement, or adherence to these behaviors encouraged by the program, was summarized by calculating the total number of activities performed by each participant over the 16 week study period: (1) meals logged, (2) weigh-ins completed, (3) exercises logged, (4) messages sent to individual coaches, (5) articles read, and (6) group activities (posts, post hearts, thread posts, thread post hearts). These six frequencies were summed to form a total engagement score.

Weight Loss. Weight was measured via self-report on the program. Weight loss was calculated by subtracting latest weight from initial weight logged within the app. Last-observation-carried-forward was used for any missing weight data. Seven participants did not log more than one weight within the app and were excluded from analyses related to weight loss.

### 2.6. Statistical Analysis

For the primary aim, paired *t*-tests compared baseline and follow-up (16 week) outcomes (i.e., body acceptance, body image flexibility, self-compassion, and rumination). To measure the relationship between changes in outcomes (from baseline to follow-up) and weight loss, unadjusted and adjusted analyses were conducted. Unadjusted Pearson correlations examined the relationship between outcome change scores (each outcome’s change score as a separate independent variable) and weight loss (dependent variable). Linear regression models then adjusted for baseline weight. For this study’s secondary aim, Pearson correlations were conducted between body positivity and self-compassion scales at baseline and the total engagement score. The same analysis was then conducted with change scores and engagement. Analyses were conducted in SPSS (version 27; IBM Corporation, Armonk, NY, USA) with an alpha of 0.05. *t*-tests were corrected for multiple tests using the Sidak–Bonferroni correction [45]. Effect sizes were measured using Cohen’s d [46]. Though interpretation varies based on the field, effect sizes are typically considered to be small (d ≥ 0.2), medium (d ≥ 0.5), or large (d ≥ 0.8) [46].

## 3. Results

### 3.1. Participant Characteristics

Participants were mainly female (*n* = 106, 80%), married (*n* = 87, 65.4%), and employed (*n* = 102, 76.7%); had a four-year college degree or more advanced education (*n* = 107, 80.4%); and identified as non-Hispanic Caucasian (*n* = 106, 79.7%). The average age was 45.38 (SD = 13.34). There were no significant group differences across participants who completed the baseline survey only versus those who completed both baseline and follow-up surveys. Weight significantly decreased from baseline to 16 week follow-up (t(128) = 10.64, *p* < 0.001, d = 0.94). Participants lost an average of 3.91 kg (SD = 4.18), with 36% achieving weight loss ≥ 5% of their initial body weight. Over 16 weeks, participants did an average of 937 in-app actions (SD = 555.91). Participants averaged 3.34 actions (SD = 1.81) per week over 16 weeks.

### 3.2. Changes in Outcomes

First, we examined if body positivity, self-compassion, and rumination changed over the course of the program (Table 1). Body appreciation and body image flexibility significantly improved from baseline to 16 weeks (t(132) = 5.23, *p* < 0.001, d = 0.45; t(132) = 3.56, *p* < 0.001, d = 0.31). Similarly, self-compassion significantly improved over the course of the program, as seen in the summary variable of total self-compassion (t(132) = 4.04, *p* < 0.001, d = 0.35), along with the subscales of self-judgment (t(132) = 3.87, *p* < 0.001, d = 0.34), common humanity, (t(132) = 3.64, *p* < 0.001, d = 0.32), and mindfulness (t(132) = 2.72, *p* = 0.007, d = 0.24). Marginally significant improvements were also observed in the subscales of self-kindness (t(132) = 1.66, *p* = 0.10, d = 0.14) and overidentification (t(132) = 1.88, *p* = 0.06, d = 0.16). No change was seen in the subscale of isolation (t(132) = −1.12, *p* = 0.27). Finally, rumination concerning food, figure, and weight improved from baseline to 16 weeks, as observed in the summary variable of total rumination (t(132) = 2.92, *p* = 0.004, d = 0.25) as well as the brooding subscale (t(132) = 3.11, *p* = 0.002, d = 0.27). There was no significant change in the reflection subscale (t(132) = 1.47, *p* = 0.14).

Next, we examined whether these changes were associated with the amount of weight participants lost. In both unadjusted correlations and adjusted regression models, weight loss was only associated with changes from baseline to 16 weeks in body appreciation ((r(127) = 0.24, *p* = 0.007; B = 0.23, *p* = 0.01, 95% CI 0.48–3.39); increased body appreciation over time was associated with greater weight loss. Weight loss was not associated with changes in self-compassion, body image flexibility, and rumination in both unadjusted and adjusted models. This suggests that these improvements were independent of weight loss.

### 3.3. Baseline Measures and Engagement

Then, we examined whether baseline measures of the outcomes were associated with engagement over the 16 week study. Baseline levels of total self-compassion, as well as the self-judgment subscale of self-compassion, were significantly associated with total engagement ((r(131) = 0.19, *p* = 0.03; r(131) = 0.24, *p* = 0.006, respectively). The higher participants’ initial levels of total self-compassion, and the lower their self-judgment, the more they engaged in relevant behaviors. No other self-compassion subscales at baseline were significantly associated with engagement. Similarly, no scales of body appreciation, body image flexibility, and rumination were significantly associated with engagement.

### 3.4. Changes in Outcomes and Engagement

Finally, we examined whether improvements in these outcomes over time were associated with engagement. Overall engagement over 16 weeks was significantly associated with improvements in body appreciation (r(131) = 0.17, *p* = 0.04) and body image flexibility (r(131) = −0.23, *p* = 0.007). Engagement was also significantly associated with the brooding subscale of rumination (r(131) = −0.23, *p* = 0.007), in which greater engagement was associated with improvement over time in brooding. No other scales were significantly associated with engagement, including overall rumination and all self-compassion subscales.

Further, we examined which specific engagement measures showed significant associations with these improvements. The frequency of coach messages, articles read, and meals logged was significantly associated with changes in body appreciation (r(131) = 0.17, *p* = 0.05; r(131) = 0.17, *p* = 0.05; r(131) = 0.23, *p* = 0.009), body image flexibility (r(131) = −0.27, *p* = 0.002; r(131) = −0.27, *p* = 0.002; r(131) = −0.24, *p* = 0.005), and the brooding scale of rumination (r(131) = −0.30, *p* = 0.001); r(131) = −0.30, *p* = 0.001; r(131) = −0.23, *p* = 0.009). The frequency of weigh-ins, exercises logged, and group interactions was not significantly associated with changes in body appreciation, body image flexibility, or brooding (all ps = n.s.). Thus, the amount of engagement in interactions with the coach, reading the articles, and logging meals were most strongly related to improvements in body positivity (i.e., body appreciation, body image flexibility, and body-related brooding). There were no significant correlations between specific engagement measures and changes in all self-compassion subscales or the other rumination subscales aside from brooding.

## 4. Discussion

In this single-arm prospective study, we sought to examine changes in body positivity and self-compassion among individuals who had self-enrolled in a digital commercial program informed by ACT, DBT, and CBT. We also explored how these measures were associated with engagement in program-measured relevant behaviors. From baseline to end of the program (16 weeks), body appreciation, body image flexibility, self-compassion (as in total self-compassion and subscales of self-judgment, common humanity, and mindfulness), and rumination (as in total rumination and the brooding subscale) significantly improved. These changes were not related to weight loss, with the exception of body appreciation. Average weight loss from baseline to 16 weeks was statistically significant but modest (3.9 kg), and 36% of participants achieved clinically significant weight loss (≥5% body weight). Baseline total self-compassion and self-judgment were associated with higher engagement, but there was no significant association for baseline body appreciation, body image flexibility, and rumination. Higher engagement was associated with improvements over time in body appreciation, body image flexibility, and rumination. In particular, more articles read, coach messages, and meal logs were associated with improvements in these outcomes.

Weight management programs tend to focus on behavioral changes that modify weight, raising questions about how and whether users experience changes in body positivity and self-compassion. To our knowledge, this is the first study to examine body positivity and self-compassion on a weight management program that is psychologically-oriented but not primarily tailored towards body image or self-compassion. This makes it difficult to directly compare our results with previous studies. However, our findings corroborate past evidence. Past prospective evidence, primarily from randomized controlled trials, suggests that standard behavioral weight management (SBW) programs or SBW programs enhanced with CBT have demonstrated improvements in body image and body dissatisfaction (see [11,27] for a review). Our results add to this literature by showing that body *positivity* and self-compassion significantly improved over time. Our findings are also consistent with studies showing improvements in body image from targeted CBT-, ACT-, or DBT-based programs, as well as programs specifically targeting self-compassion [21,22,23,47,48]. Our results also contribute new knowledge by showing that changes in body positivity and self-compassion can occur in individuals who voluntarily joined and used a digital publicly available program. This raises additional directions for future research about the similarities between this population and those recruited to participate in controlled trials. Future studies should directly compare body positivity and self-compassion in this population and those in controlled settings, as well as qualitatively understand body positivity or self-compassion concerns from this population.

The non-significant associations between weight loss and changes in body image flexibility, body-focused rumination, and self-compassion suggest that these improvements did not occur because participants’ weight or bodies changed. This departs from previous studies of SBW programs finding that weight loss mediated the effect of the intervention on improved body image [26,49]. Future research should investigate the possibility that this is because ACT, DBT, and CBT-based programs help improve body positivity and self-compassion in ways that do not depend on losing weight or body size changes. This aim is inherent in these psychological frameworks but could not be directly tested in this study. We found a significant association between weight loss and improvements in body appreciation, which could be due to enhanced engagement. Better engagement would help individuals increase appreciation for their bodies through the curriculum and is strongly associated with greater weight loss [32,49]. On the other hand, with the present study, we could not rule out the possibility that increased body appreciation stemmed from weight loss enabling participants’ bodies to look closer to their ideal body type, which may not ultimately indicate appreciation of one’s body no matter what. However, while individuals lost significant weight compared to baseline, this amount of weight loss (3.91 kg) is unlikely to lead to substantial changes to body size, and there were significant improvements in body image flexibility that were not related to weight loss. Future research should tease apart the contribution of weight loss to body appreciation and ideal body sizes in this population and type of program with causal designs and methods.

The finding that baseline self-compassion was associated with greater overall engagement is consistent with empirical and theoretical work. With self-compassion, individuals are better able to show themselves kindness and understanding for their limitations and hardships, which may generate increased motivation to persist in valued behaviors even after initial setbacks [50,51]. Self-compassion has been linked to greater intrinsic motivation, resilience, and persistence [8,50,52]. Our findings add to this knowledge by raising the question of whether initial self-compassion is more important for engagement than over-time improvements; perhaps those who have the highest underlying self-compassion and the lowest levels of self-judgment engage most. Engagement was not associated with changes in self-compassion (including self-judgment), which suggests that changes in self-compassion occurred similarly regardless of level of engagement. At the same time, greater total weekly engagement was associated with improvements in the brooding component of rumination. Brooding includes repeating negative thoughts about why one should have done better, which is more oriented towards behaviors or actions (“why do I always react this way around food?”) than self-judgment more generally (“I’m disapproving and judgmental about my own flaws and inadequacies”). Therefore, brooding is associated with avoidance behaviors and impaired problem solving [53,54]. Taken together with our results, this suggests that engagement behaviors could help improve brooding more than self-judgment or other aspects of self-compassion. Future research should test this potential explanation. Since these were correlational analyses, future research should also test the directionality of these relationships.

Total weekly engagement was also associated with greater improvements over time in body appreciation and body image flexibility. Not all types of program engagement were associated with these over-time improvements. In particular, the engagement measures most strongly associated with improvements in body positivity were coach messages, articles, and meal logging. In this program, the ACT, DBT, and CBT content was mostly delivered through the articles and coach interactions. This suggests the possibility that engaging more in ACT, DBT, and CBT content, even within a weight loss program, can motivate a more positive body perception. These results build on previously explained studies indicating that exposure to ACT-, DBT-, or CBT-based programs can improve body image [21,22,23]. Engagement in coach messages, articles, and meal logging were also associated with improved rumination in terms of brooding. Taken together, our results align with theoretical understanding that ACT and DBT encourage growing awareness of internal distress, along with acceptance and validation of these thoughts and emotions towards value-based action, and CBT encourages awareness and restructuring of negative thought patterns [15,18,36]. Our results also suggest that, at the very least, greater engagement in a weight management program is not always tied to negative changes in body image, as some have assumed, when psychologically-driven content is presented [12,55]. These results raise the implication that ACT, DBT, and CBT, when presented and interacted with in daily articles and in coach conversations, could improve body image flexibility and body-focused rumination over time to a greater extent. The interactivity of articles also could have been helpful, particularly in writing down and then challenging negative thoughts particular to the user in CBT articles, or in learning and applying the concepts of ACT and DBT through quizzes included in articles. Future research should investigate to what extent interactivity helps to improve outcomes.

Our results for self-compassion also align with the theoretical principles behind ACT, DBT, and CBT. While greater engagement was not associated with *changes* in self-compassion, we found that overall, not only did total self-compassion improve over 16 weeks, but the subscales of self-judgment, common humanity, and mindfulness showed the most improvement. These subscales concern decreasing judgment and blame towards oneself, as well as increasingly feeling that one’s distress is shared by others and that one can be mindfully aware of emotions, sensations, and thoughts. These components are directly relevant to principles in ACT, DBT, and CBT that encourage acceptance, and not blame, for feelings and thoughts; that help individuals to realize that distress and imperfection are more common than they think; and that encourage individuals to build mindful awareness and acceptance of current sensations and emotions. This suggests that articles and coaching can improve self-compassion in self-judgment, common humanity, and mindfulness when based on principles of ACT, DBT, and CBT within a generalized (i.e., not tailored towards mental wellness) weight management program. 

Additionally, meal logging was positively associated with improvements in body positivity, while weigh-ins and exercise logging were not significantly associated with changes in body positivity. These results provide important data to the literature which is currently mixed in regard to whether behaviors like self-monitoring are tied to harmful body-oriented attitudes or eating behaviors [55,56]. Our results are consistent with a recent review showing that self-monitoring may have no relationship or a positive relationship with harmful body-related attitudes or behaviors among adults with obesity seeking treatment, in contrast to populations more at risk for body dissatisfaction or disordered eating [56]. Our results go further to show a potentially positive effect of self-monitoring of food. In addition to measuring a dose effect of ACT-, DBT-, or CBT-based curricula, future research should clearly and comprehensively lay out the individual effects and directionality of health behaviors, whether measured by a program like this or not, and body positivity. Additionally, a study of a generalized health education program found reciprocal relationships between body image and weight loss where weight loss improved body image and changes in body image then improved weight loss [26]. It is possible that the correlations we found also represented cyclical relationships in which improvements in body positivity also led to further future engagement. Along these lines, a study found that improvements in body appreciation predicted increased intrinsic motivation [8]. Future work should do time-lagged analyses to better differentiate these relationships. Though we cannot definitively state directionality, our findings show there is a clear correlation between engagement, especially in psychologically-derived content, and improvements over time in body positivity. Our findings raise empirical and clinical implications, such as the possibility that emphasizing more engagement, especially exposure to an acceptance-based curriculum, could lead to greater improvements in body positivity. Future research should test this possibility.

Limitations. This study is an exploratory observation of individuals who joined a publicly available program. Because of this goal, a control group was not used, since these individuals would need to be recruited and would not provide insight into the population of interest. Thus, our findings are correlational and causal claims cannot be made. Additionally, it is impossible to rule out the possibility that improvements in body positivity and self-compassion were due solely to the passage of time. Nevertheless, our results suggest that body positivity and self-compassion do not decrease when individuals use a program that involves weight management. This is an important finding because some researchers have voiced concerns based on the assumption that any emphasis on weight management will impair body image or self-compassion [57,58]. This assumption has yet to be thoroughly tested and this study cannot provide a definitive answer, but our results suggest that it is not always true and that individuals’ body positivity and self-compassion do not deteriorate, but actually can improve, during weight management guided by ACT, DBT, and CBT. Future studies should test causal pathways being weight management components and body positivity. Another limitation is that one self-selected sample was used, which means that results may not generalize to all individuals who choose to join a digital commercial program. In addition, only four month outcomes were assessed. Future studies will investigate longer-term changes in body positivity, self-compassion, and weight loss. Finally, this study does not account for the wider complex environment surrounding individuals and their body- or self-related perceptions, such as social interactions and potentially ensuing weight stigma, or media which can elevate certain body types over others [59,60]. A full discussion is outside the scope of this study. Taking into account these external factors, some researchers encourage increased psychoeducation on weight stigma, as well as educating individuals as to what “normal” weights may actually mean [59]. Our future studies will explore these factors, such as individuals’ expectations and perceptions of “normal” weights on this type of program.

## 5. Conclusions

This study provides rare data into how body positivity and self-compassion change during real-world use of a psychologically-derived commercial weight management program outside of a controlled trial. To investigate this question, we leveraged a unique dataset and also explored how real-world program engagement relates to baseline and changes in body positivity and self-compassion. Individuals self-motivated to join a commercial program with techniques from ACT, DBT, and CBT experienced improved body positivity and self-compassion and lost modest weight. Given previous work, this could be because ACT, DBT, and CBT can help individuals to accept and integrate negative thoughts and feelings about their bodies or themselves, channeled into valuable action and self-compassion; future research should use controlled trials to investigate this question more deeply. Greater engagement in program-measured health behaviors was associated with changes in body image flexibility, body appreciation, and brooding; baseline self-compassion was also associated with greater engagement. Specifically, reading the curriculum, interacting with coaches, and logging meals were associated with greater improvements in body appreciation, body image flexibility, and brooding. This suggests that engaging with ACT-, DBT-, and CBT-based content, such as through curriculum and coach messaging, could further improve body positivity, which should be confirmed with future research. These findings have implications for how engagement with psychologically-derived content could be emphasized to improve body positivity, as well as how baseline self-compassion could be targeted to improve engagement. This study raises pertinent new directions for future research about the relationships between body positivity, self-compassion, and engagement in health behaviors; the causal role of ACT, DBT, and CBT on generalized (i.e., non-specialized) programs; and body positivity and self-compassion in this population and setting. Future work from weight management programs should also measure body positivity more as an outcome and test the effects of incorporating components of ACT, DBT, and CBT into curriculums to help individuals recognize that they can increasingly appreciate their bodies and themselves.

## Figures and Tables

**Table 1:** Change in primary outcomes from baseline to week 16.

Outcome	Mean Difference (Week 16—Baseline)	Test Statistic	*p*-Value	Cohen’s d
Body appreciation	+0.22	t(132) = −5.23	<0.001 ***	0.45
Body image flexibility ^1^	−2.86	t(132) = 3.56	0.001 ***	0.31
Total self-compassion	+2.22	t(132) = −4.04	<0.001 ***	0.35
Self-judgment subscale ^2^	+0.56	t(132) = −3.87	<0.001 ***	0.34
Common humanity subscale	+0.56	t(132) = −3.64	<0.001 ***	0.31
Mindfulness subscale	+0.39	t(132) = −2.71	0.007 **	0.24
Self-kindness subscale	+0.24	t(132) = −1.66	0.10 +	0.14
Isolation subscale ^2^	+0.19	t(132) = −1.12	0.27	0.10
Overidentification subscale ^2^	+0.29	t(132) = −1.88	0.06 +	0.16
Total rumination	−0.95	t(132) = 2.92	0.004 **	0.25
Brooding subscale	−0.71	t(132) = 3.11	0.002 **	0.27
Reflection subscale	−0.24	t(132) = 1.47	0.14	0.13

^1^ The body image flexibility scale is reverse coded such that higher scores denote worse body image flexibility. *** *p* < 0.001, ** *p* < 0.01, + *p* < 0.10. ^2^ These subscales are reverse-scored so that higher scores mean *less* self-judgment, isolation, and overidentification.

## Data Availability

Restrictions apply to the availability of these data. Data were obtained from Noom and are available by request from the corresponding author with the permission of Noom.
